# Wrist muscle performance in students with chronic nonspecific neck pain: an isokinetic assessment

**DOI:** 10.1186/s12891-026-09730-z

**Published:** 2026-03-18

**Authors:** Mohamed Mahmoud Refaey, Enas Fawzy Youssef, Aness Saleh Gheit, Mohamed Abdelaziz Emam, Aya Abdelhamied Mohamed Khalil

**Affiliations:** 1 Biomechanics Department, Faculty of Physical Therapy, Kafr El Sheikh University, Kafr El-Shaikh, Egypt; 2 Department of Musculoskeletal Disorders and its Surgeries, Faculty of Physical Therapy, Cairo University, Cairo, Egypt; 3 Basic Sciences Department, Faculty of Physical Therapy, Kafr Elsheikh University, Kafr El-Sheikh, Egypt; 4 János Szentágothai Neurosciences Division, Semmelweis University, Budapest, 1085 Hungary; 5 Biomechanics Department, Faculty of Physical Therapy, Cairo University, Cairo , Egypt

**Keywords:** Chronic nonspecific neck pain, Upper extremity biomechanics, Wrist flexors and extensors, Isokinetic dynamometry, Peak torque, Muscle endurance, Neuromuscular performance

## Abstract

**Background:**

Work-related musculoskeletal disorders (WMSDs) are highly prevalent among physiotherapy students and therapists. This high prevalence highlights the specific link between sustained cervical loading which can precipitate nonspecific neck pain and upper extremity dysfunction, clarifying that proximal neck pain may contribute to distal upper extremity impairment. This observational cross section comparative study investigated whether chronic nonspecific neck pain (CNSNP) influences wrist flexor and extensor performance metrics, such as peak torque, average power, total work and work fatigue.

**Methods:**

Ninety participants (*n* = 36 females, *n* = 54 males, age: 18–23) were allocated to either the CNSNP group (*n* = 44 participants with CNSNP) or the asymptomatic control group (*n* = 46 participants without CNSNP). A Biodex isokinetic dynamometer operating at an angular velocity of 180°/s was used to measure the endurance parameters of the wrist flexors and extensors.

**Results:**

Chronic nonspecific neck pain did not significantly affect wrist muscle performance parameters (peak torque, average power, total work, work fatigue) measured by isokinetic dynamometry at 180°/s (F (1,86) = 1.40, *p* = 0.241, η²partial = 0.06). While there was no difference in muscle performance across groups, there was a significant difference in flexion and extension movements (F (1,86) = 7.86, *p* < 0.001, η²partial = 0.27). Wrist flexion peak torque and pain intensity showed a positive correlation (*r* = 0.47, *p* = 0.03), but no other significant correlations were found.

**Discussion and conclusions:**

CNSNP did not significantly impair overall wrist muscle performance in these young physiotherapy students and interns, revealing preserved neuromuscular capacity and movement‑specific modulation of wrist motor control rather than a global loss of force output, clarified by the pain-related increase in wrist flexor peak torque.

**Clinical trial registration:**

ClinicalTrials.gov Identifier NCT06240611 (February 5, 2024) Retrospectively registered.

## Background

A common orthopedic condition is nonspecific neck pain, which is defined by uncomfortable neck symptoms and limited mobility in the absence of clinical or structural abnormalities [1]. According to research, medical college students frequently experience discomfort in their necks. Furthermore, some teenagers complain of related hand and wrist pain [2]. Persistent lower back pain is more common in men [3], while wrist numbness, neck, and shoulder pain are more common in women [4].

Chronic nonspecific neck pain (CNSNP) has negative functional effects in addition to causing local pain. Reduced upper limb muscle function is strongly correlated with CNSNP, which may lead to impairment [5]. CNSNP can negatively impact upper limb strength, endurance, and proprioception – all of which are necessary for daily tasks [6]. Neck discomfort has been linked to abnormal muscle function, impaired proprioception, and joint instability [7]. Position perception and upper limb movement are further complicated by these problems [8].

Hand dysfunction and CNSNP are strongly correlated in earlier research using validated instruments [9, 10]. Few studies, however, have explicitly examined how CNSNP affects physiotherapy students’ functional abilities in the wrist and upper extremities [11–14]. Furthermore, because few studies have looked at muscle endurance involving concentric muscle activities in this context, there is a knowledge gap regarding the wider effects of CNSNP on upper limb impairment.

The selection of this specific population was highly intentional due to the unique biomechanical and occupational demands of their academic and clinical training [15]. Unlike the general student population, Physiotherapy students are regularly engaged in extensive practical sessions and manual therapy training. These activities require sustained, awkward cervical postures combined with repetitive, forceful use of the upper extremities and wrists, such as applying pressure and performing joint mobilizations [16]. This dual physical loading makes them a highly susceptible and clinically relevant population for investigating the neuro-kinematic link between chronic cervical pain and distal motor performance.

This study examined the effects of CNSNP on wrist function among Egyptian Physiotherapy students and interns to address these disparities. It is expected that the results would set new benchmarks for evaluating hand function in physiotherapy patients with neck pain and help in the foundation of focused treatments for these demographics.

## Methods

### Aim of the study

This study aimed to evaluate wrist extensor and flexor performance parameters (peak torque, average power, total work, work fatigue) among Egyptian Physiotherapy students and interns with chronic nonspecific neck pain. It was conducted at the isokinetic dynamometry laboratory at the Faculty of Physical Therapy, Cairo University, Cairo, Egypt, from May 2023 to December 2023.

### Study design and setting

This observational cross-sectional comparative study utilized an isokinetic dynamometer to assess the performance of wrist extensors and flexors (peak torque, average power, total work, work fatigue) in concentric contraction mode at a speed of 180°/s.

### Ethics approval

The study was approved by the Institutional Review Board of the Faculty of Physical Therapy, Cairo University (protocol code P.T.REC/012/004563 and date of approval 7 May 2023) and registered retrospectively on ClinicalTrials.gov (Registration Number: NCT06240611 and date of approval 5 February 2024) Retrospectively registered. Participants were recruited over six months and provided informed consent for participation and publication.

### Participants

**Fig. 1:**
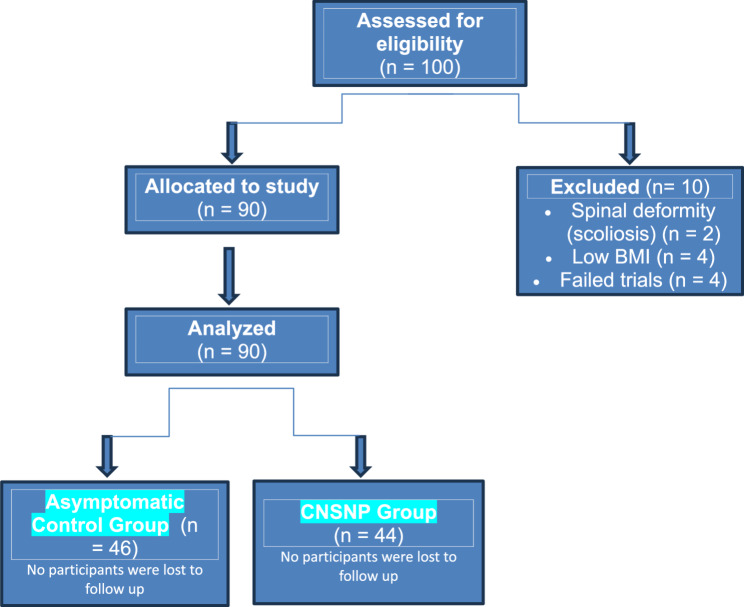
STROBE Flow chart diagram of the current study

Participants consisted of active physiotherapy students and interns recruited from the University Outpatient Clinics during their mandatory clinical training rotations. Participants were approached via (inclusion announcements and direct call) and were assigned into two groups: Group A (asymptomatic control group) and Group B (CNSNP group). The sample size was calculated using the G Power test method (Fig. 1).

The inclusion criteria for the CNSNP group were: (1) students of both sexes between the ages of 18 and 23 years [9]; (2) orthopedic surgeon-diagnosed CNSNP lasting longer than three months without neurological symptoms [17]; (3) a body mass index (BMI) between 18.5 and 29.5 [18]; and (4) neck pain-related disability, as defined by a score of 5–14 points (10–28%) on the Neck Disability Index (NDI) and pain intensity between 3 and 8 on the Numerical Pain Rating Scale (NPRS). As for participants in the asymptomatic control group, they were with comparable BMIs and age and had no prior history of neck pain within the past 12 months [19].

Exclusion criteria included: (1) neurological disorders such as stroke, Parkinson’s disease, peripheral neuropathy, or multiple sclerosis [20]; (2) diagnosed headache; (3) history of cancer, infection, or other systemic illnesses; (4) recent neck, shoulder, and head trauma in the last year, or any traumatic spinal cord injury [21]; (5) cardiovascular or cerebrovascular disorders; (6) musculoskeletal disorders (e.g., joint replacement, arthritis, or muscular dystrophy) [22]; (7) prior upper limb or hand surgery [9]; (8) cervical spine issues (e.g., spondylosis, disc prolapse, or fractures); (9) recent neck pain treatment within the last three months [21]; (10) sensory system conditions (e.g., visual, auditory, or speech problems); 11) History of orthopedic or neurological conditions such fractures, surgeries on the upper limb or hand, carpal tunnel syndrome, De Qurvain’s syndrome, or diabetic mellitus that result in functional defects of the hand strength [9], and 12) Participants were excluded if they presented with forward head posture, ass assessed by a Craniovertebral Angle (CVA) of < 50° [23].

### Instruments

### The isokinetic dynamometer for measuring endurance parameters

**Fig. 2:**
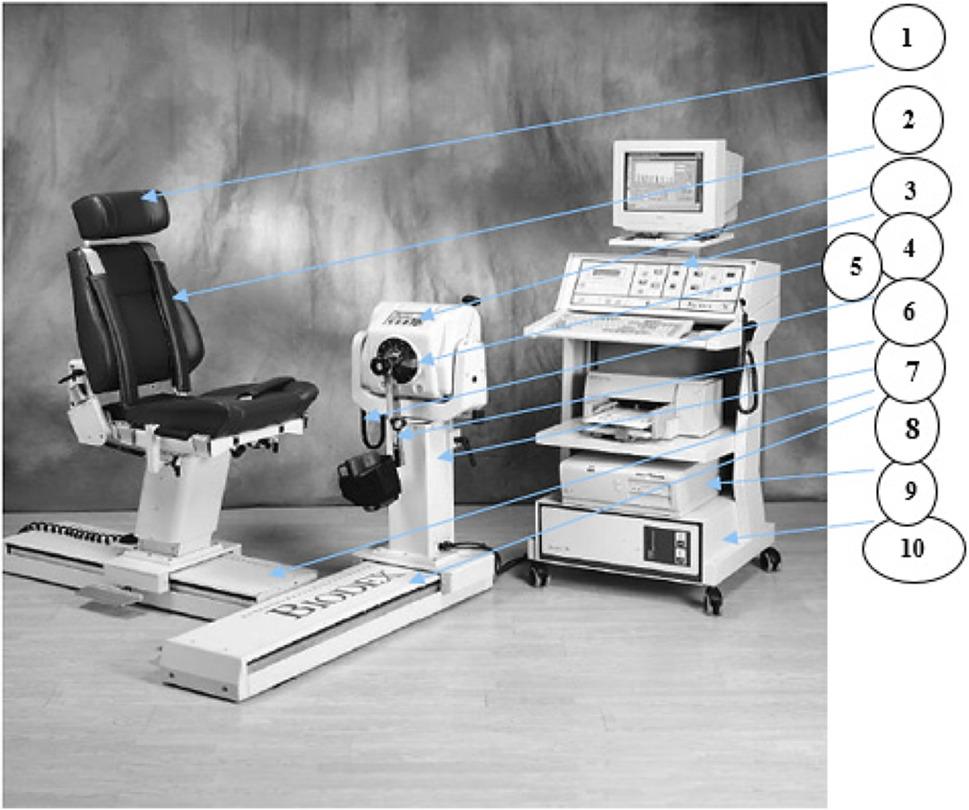
Biodex isokinetic dynamometer system 3 pro®. (1) Seat. (2) Securing straps. (3) Emergency stop. (4) Machine control. (5) Motion limit stop. (6) Comfort stop switch. (7) Actuator arm. (8) 3 Movable arms. (9) Isolated power supply. (10) Computer (Adopted from Manual of Biodex Medical Systems)

The isokinetic dynamometer (Biodex System 3 PRO^®^) was utilized to assess concentric wrist flexor and extensor muscle endurance at an angular velocity of 180°/s for both groups. Before testing, the dynamometer was calibrated to ensure accurate measurements. All isokinetic tests, which are valid, reliable, and responsive to assess outcomes in participants with CNSNP, were performed by the same evaluator [24] (Fig. 2).

### Kinovea software for measuring craniovertebral angle

**Fig. 3:**
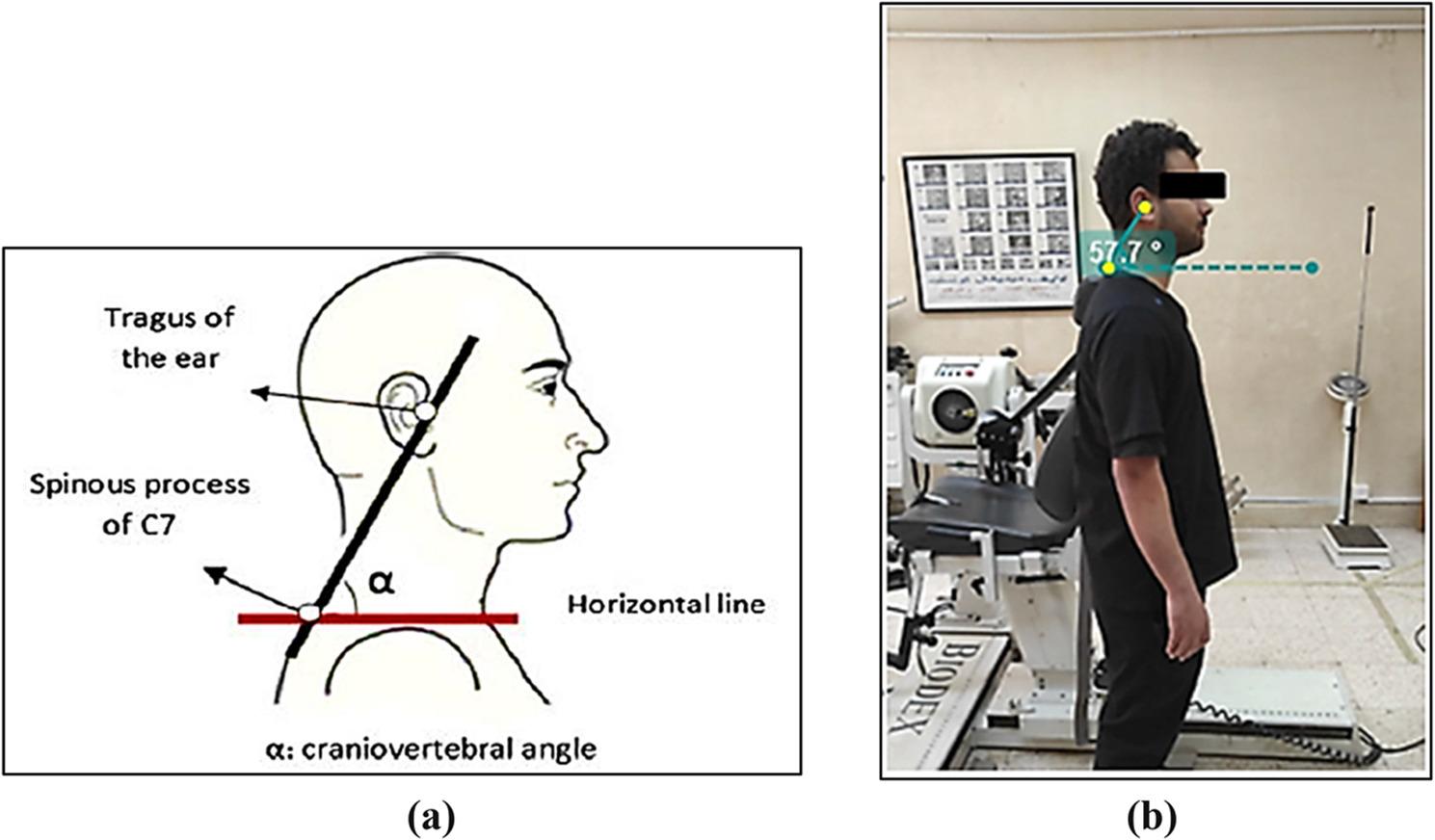
Kinovea Software for measuring craniovertebral angle; (**a**) Reference markers on C7 spinous process and tragus of ear; (**b**) Kinovea software assessing forward head posture by measuring cranio-vertebral angles

When evaluating cervical range of motion in the sagittal plane, the Kinovea software tool has demonstrated both interrater and intrarater reliability. It offers a respectable degree of precision for linear and angular measurements made by digitizing x- and y-axis coordinates [25, 26]. In this study, participants with a cervical vertebral angle (CVA) of 50° or greater were included, while those with a CVA below 50° were excluded, as per the criteria established by [27] (Fig. 3).

### Numerical pain rating scale for evaluating pain intensity

**Fig. 4:**
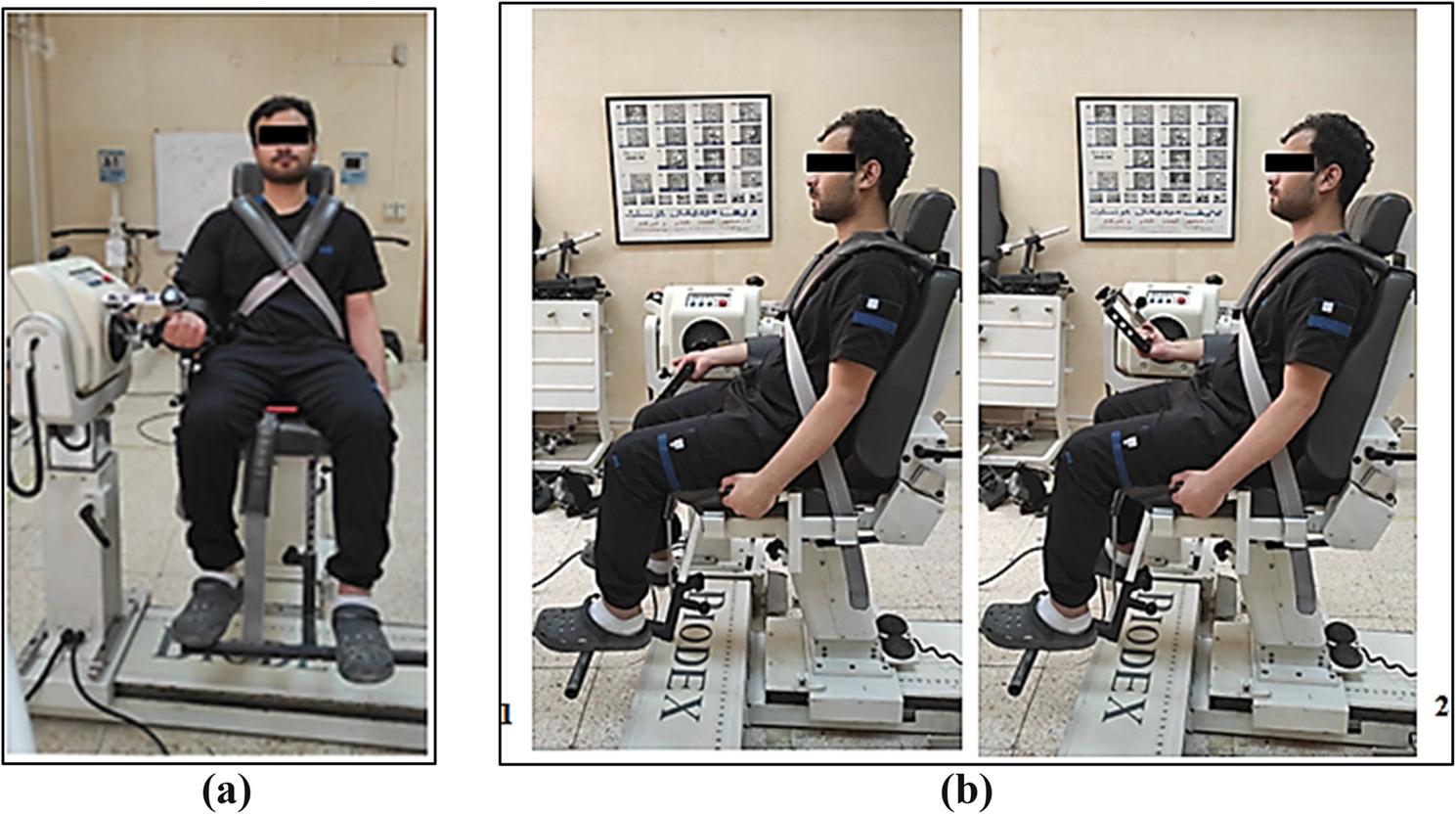
Biodex isokinetic dynamometer for wrist assessment: (**a**) Frontal view; (**b**) Sagittal view; (1) Extension direction; (2) Flexion direction

The Numerical pain rating scale (NPRS) was used to measure the intensity of pain. This scale is considered a valid, reliable, and responsive method for assessing pain in patients with CNSNP [28]. NPRS is a validated patient-reported outcome measure with established reliability (ICC = 0.67–0.88) and construct validity (*r* = 0.86–0.95 with VAS) in chronic neck pain populations [29]. According to the study criteria, the pain intensity reported by the participants ranged from 3 to 8 on this scale (Figure 4.)

### Arabic version of the neck disability index for evaluating neck pain-related disability

The Neck Disability Index (NDI), a widely used and validated instrument for assessing functional impairment in patients with neck pain, was used to assess the level of disability in patients with CNSNP. The NDI-AR is a reliable, widely used, and highly adapted instrument in the Arabic population, with an excellent intraclass correlation coefficient (ICC) of 0.96 [30]. The test-retest reliability of the NDI is ICC = 0.88, and its validity is ICC = 0.86 [31]. According to the study criteria, the pain-related disability reported by the participants ranged from mild to moderate disability on this index.

### Procedure

A convenient sample of ninety participants who provided consent forms after receiving a brief orientation session about the nature of the study, the study purpose, and procedure, was selected. Then, their individual demographic data was recorded in data sheet including participant age, gender, weight, height, and pain intensity.

Before testing, the dynamometer was calibrated, and participants were familiarized with the device by performing three submaximal concentric wrist extension and flexion trials. To eliminate the confounding effects of motor learning and muscular fatigue on the testing day, all participants underwent a dedicated familiarization session on the isokinetic dynamometer [24 to 48 h] prior to their formal experimental testing. This temporal separation between familiarization and data collection aligns with established methodological guidelines [32]. All isokinetic assessments were performed on the wrist of the participant’s dominant upper extremity. Hand dominance was established by asking the participant their preferred hand for writing/eating. During the testing protocol, the order of wrist movements was (e.g., flexion followed by extension) for all participants.

Performance parameters of wrist extensors and flexors, including peak torque, average power, total work and work fatigue, were evaluated using a Biodex System 3 isokinetic dynamometer^®^. These parameters were measured for every participant in the sitting position with the back of the seat tilted to 90 degrees, while flexing their hip and knee joints to 90 degrees. The dynamometer’s rotational axis was aligned with the radius at the radiocarpal joint, the capitate bone, and the carpal bones in the proximal row. Four securing belts were used: two across the trunk, one around the pelvis, and the last around the wrist to stabilize the participant [33].

To evaluate performance, participants performed fifteen maximal concentric contractions at a speed of 180°/s, within a range of motion of 85 degrees (45 degrees of wrist flexion and 40 degrees of wrist extension). A one-minute rest period was provided between each trial. Limb weight was measured before each test, and torque data were adjusted by the data acquisition software to account for the effect of gravity [34]. An angular velocity of 180°/s was selected as it is the established isokinetic standard for evaluating muscular performance parameters (peak torque, average power, total work, work fatigue) [35, 36]. Higher testing velocities facilitate the rapid, repetitive contractions necessary to tax local muscular endurance pathways while minimizing excessive joint compressive and shear stress at the radiocarpal joint [37].

Work fatigue was calculated by the Biodex software as the percentage of decline in total work between the first third (repetitions 1–5) and the final third (repetitions 11–15) of the 15-repetition isokinetic endurance protocol ignoring the middle repetitions (6–10) to isolate the performance drop-off, using the formula: Work Fatigue (%) = [(Work of first 5 reps − Work of last 5 reps) / Work of first 5 reps] × 100.

### Statistical analysis

Statistical analysis was performed using R (version 4.5.1; R Foundation for Statistical Computing, Vienna, Austria). Data were presented as mean ± standard deviation (SD). Normality of the data was assessed using the Shapiro-Wilk test. Descriptive statistics were applied to analyze the general characteristics of the participants. To compare subject characteristics between the two groups, an unpaired t-test was conducted. A two-way multivariate analysis of variance (MANOVA) was conducted using the (MANOVA) function to examine the effects of group (between-subjects: CNSNP vs. asymptomatic control) groups and condition (within-subjects: flexion vs. extension) on the combined set of four dependent variables: peak torque, total work, work fatigue, and average power. Pillai’s trace was used as the multivariate test statistic.

For multivariate effects, Pillai’s trace (V) was reported as the effect size measure, as it directly represents the proportion of multivariate variance explained by each effect. For univariate effects, partial eta-squared (η²p) was calculated as SS effect / (SS effect + SS error) using the (eta_squared) function from the effect size package (version 1.0.1) with the partial = TRUE argument. Following Cohen’s (1988) guidelines, η²p values of 0.01, 0.06, and 0.14 represent small, medium, and large effects, respectively [38]. The significance level was set at *P* ≤ 0.05.

Following significant multivariate effects, univariate ANOVAs were conducted for each dependent variable as follow-up tests using the (summary.aov) function. Post-hoc pairwise comparisons were performed using estimated marginal means (EMMs) with Bonferroni adjustment for multiple comparisons. The significance level was set at *P* ≤ 0.05. To address the between-group difference in BMI (*p* = 0.045), sensitivity analyses using analyses of covariance (ANCOVA) were performed with the (ANOVA) function from the car package (version 3.1.3), with BMI included as a covariate for each dependent variable. The ANCOVA models examined the main effects of group and condition, as well as their interaction, while controlling BMI.

Sample size calculation using G*Power software (version 3.1.9.7; Franz Faul, Universität Kiel, Germany) confirmed that eighty-five participants would provide adequate statistical power for the planned analyses. The calculation was based on an F-test for MANOVA: Repeated measures, within-between interaction. We used an effect size (f(V)) of 0.36, which was derived from a previous study by Agirman et al. (2017) [39] that assessed peak torque of the wrist using an isokinetic dynamometer. The calculation assumed the following parameters: alpha level (α): 0.05, power (1-β): 0.80. Based on these inputs, the minimum required total sample size was calculated to be 89 participants. To account for a potential dropout rate of approximately 15%, we recruited a total of 100 participants (50 in each group) (Fig. 2).

All participants included in the final analyses completed the full testing protocol, and there were no missing data across any of the key variables, including demographics, clinical measures (NPRS, NDI), CVA, and all isokinetic outcomes.

## Results

### Participant characteristics

This study recruited ninety physiotherapy students and interns, with forty-six participants assigned to the asymptomatic control group and forty-four to the CNSNP group. The sample consisted of fifty-four males (60%) and thirty-six females (40%), with demographic characteristics presented in Table 1. As for participants in the asymptomatic control group, participants were recruited to be comparable in age and BMI; however, groups were not formally matched. Before analysis, normality testing confirmed that all dependent variables were normally distributed (Table 2).

**Table 1:** Descriptive characteristics of participants by group

	CNSNP Group	Asymptomatic Control Group	p-value
	(n = 44)	(n = 46)	
Female, n (%)	16 (36.4%)	22 (47.8%)	0.375
Male, n (%)	28 (63.6%)	24 (52.2%)	0.375
Age (years)	20.59 (1.72)	20.70 (1.77)	0.777
Body mass (kg)	72.18 (12.44)	68.87 (10.42)	0.176
Height (m)	1.71 (0.10)	1.71 (0.08)	0.781
BMI (kg/m²)	24.73 (3.15)	23.53 (2.37)	0.045

Values are mean (SD) unless otherwise indicated. p-values for categorical variables were calculated using chi-square tests; for continuous variables, independent t-tests were used

**Table 2:** Normality test for all dependent variables

	Work		Torque		Fatigue		Power	
	Flexion	Extension	Flexion	Extension	Flexion	Extension	Flexion	Extension
Asymptomatic Control Group(Gr. A)	0.200	0.119	0.108	0.341	0.060	0.682	0.182	0.112
CNSNP Group (Gr. B)	0.819	0.200	0.051	0.636	0.051	0.805	0.229	0.483

Shapiro–Wilk test p-values; p > 0.05 indicates data are normally distributed

### Multivariate results

A multivariate ANOVA was conducted to examine the effects of group membership (CNSNP vs. asymptomatic control) groups and muscle contraction condition (flexion vs. extension) on four dependent variables: peak torque, average power, total work and work fatigue.

Using Pillai’s trace criterion, the multivariate main effect of group was not statistically significant, Pillai’s V = 0.063, F (4, 83) = 1.40, *p* = 0.241. This indicates that when comparing students with CNSNP to asymptomatic control group across all four outcomes simultaneously, no significant between-group difference was observed in overall muscle performance. The effect size (V = 0.063, representing 6.3% of multivariate variance) was small according to conventional benchmarks. However, a significant multivariate main effect of condition emerged, Pillai’s V = 0.275, F (4, 83) = 7.86, *p* < 0.001. This demonstrates that when comparing flexion to extension movements across all participants (regardless of group), muscle performance differed significantly between these two conditions. The effect size (V = 0.275, representing 27.5% of multivariate variance) was large, indicating a substantial difference in muscle performance between the two movement conditions.

The multivariate interaction between group and condition was not statistically significant, Pillai’s V = 0.027, F (4, 83) = 0.57, *p* = 0.683, indicating that the pattern of differences between flexion and extension was similar in both the CNSNP and asymptomatic control groups. The minimal effect size (V = 0.027, representing 2.7% of multivariate variance) confirmed the absence of a meaningful interaction.

It is important to note that while the multivariate analysis revealed no overall group effect or significant group × condition interaction, exploratory examination of individual outcomes in specific conditions (as detailed in Table 3) may reveal condition-specific patterns. However, such patterns should be interpreted cautiously in the absence of a significant multivariate group effect or interaction, as they represent exploratory findings that may not generalize across the broader set of.

outcomes. The following univariate and descriptive analyses examine each dependent variable individually to identify potential condition-specific patterns warranting further investigation.

### Follow-up univariate analyses

Given the significant multivariate condition effect, post hoc univariate ANOVAs were conducted to identify which specific variables contributed to this finding. Among all dependent variables, only peak torque demonstrated a significant condition effect F (1,86) = 4.74, *p* = 0.032, η²*p* = 0.052, indicating a moderate difference between flexion and extension movements.

### Post-hoc and exploratory condition-specific comparisons

Descriptive statistics and between-group comparisons for all measured variables across flexion and extension conditions are presented in Table 3.

**Table 3:** Descriptive statistics and between-group comparisons of wrist muscle performance during flexion (FLEX) and extension (EXT) in students with chronic non-specific neck pain (EXP) and asymptomatic control (CON) groups

	FLEXMean ± SD	EXTMean ± SD	MD(within-group)	p-value(within-group)
AVERAGE_POWER_(W)				
EXP	4.99 ± 3.18	5.22 ± 3.55	0.24	0.817
CON	5.98 ± 4.50	7.37 ± 3.54	1.39	0.252
MD	0.99(p = 0.397)	2.14(p = 0.049)		
PEAK TORQUE (N.M)				
EXP	6.00 ± 2.37	6.75 ± 2.60	0.74	0.329
CON	6.28 ± 3.07	7.94 ± 2.42	1.66	0.035
MD	0.28(p = 0.735)	1.19(p = 0.119)		
TOTAL WORK (J)				
EXP	51.65 ± 32.96	60.59 ± 41.46	8.93	0.434
CON	63.56 ± 42.18	84.00 ± 35.96	20.45	0.084
MD	11.90(p = 0.296)	23.42(p = 0.050)		
WORK FATIGUE (%)				
EXP	6.39 ± 48.35	-22.50 ± 129.39	-28.89	0.335
CON	4.14 ± 26.71	4.96 ± 25.93	0.81	0.917
MD	-2.25(p = 0.849)	27.45(p = 0.339)		

*MD* Mean difference, *SD* Standard deviation, Within-group p-values were calculated using paired t-tests comparing flexion vs. extension. Between-group p-values were calculated using independent t-tests comparing EXP vs. CON for each condition.

All primary analyses were prespecified in the study protocol and focused on the multivariate effects of group (CNSNP vs. asymptomatic control) and condition (flexion vs. extension) on four main outcomes: peak torque, average power, total work, work fatigue. Post hoc univariate comparisons for specific conditions (e.g., extension-only differences or trends) were conducted to explore condition-specific patterns revealed by significant multivariate effects. For post hoc analyses, estimated marginal means (EMMs) with Bonferroni-adjusted pairwise comparisons were applied to asymptomatic control group for multiple testing. All condition-specific trends that did not survive this adjustment are interpreted as exploratory.

### Average power

**Fig. 5:**
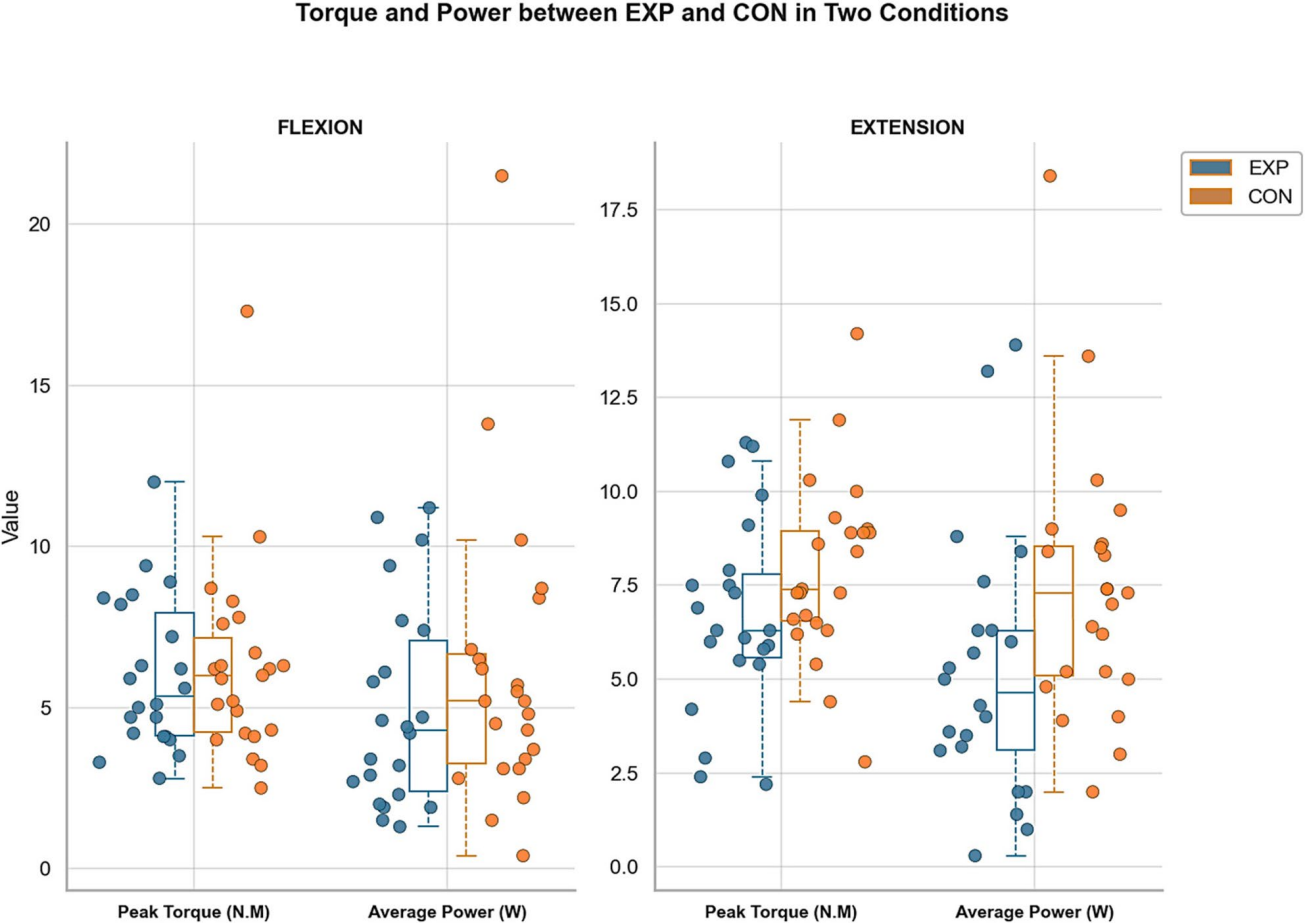
Comparison of average torque and power between Asymptomatic control and CNSNP groups during wrist flexion and extension. Note: Students with chronic non-specific neck pain (EXP) and Asymptomatic control (CON) group

Both groups demonstrated slightly higher average power during extension compared to flexion movements. A significant between-group difference was observed only during extension (*p* = 0.049, between-group), indicating a condition-specific between-group difference under the extension condition. This difference should be interpreted cautiously, as it emerged from a condition-specific post hoc comparison (Fig. 5).

### Peak torque

Peak torque values consistently favored extension over flexion across both groups. In the asymptomatic control group, participants generated 1.66 N·m more torque during extension, a statistically significant within-group difference (*p* = 0.035, within-group). Mean peak torque across groups was significantly higher during extension compared to flexion in post hoc analyses with Bonferroni adjustment (t(88) = 2.17, *p* = 0.033, Cohen’s d = 0.46), indicating a moderate effect size. These findings highlight a condition-specific strength difference while maintaining that overall multivariate group effects were non-significant (Fig. 5).

### Total work

**Fig. 6:**
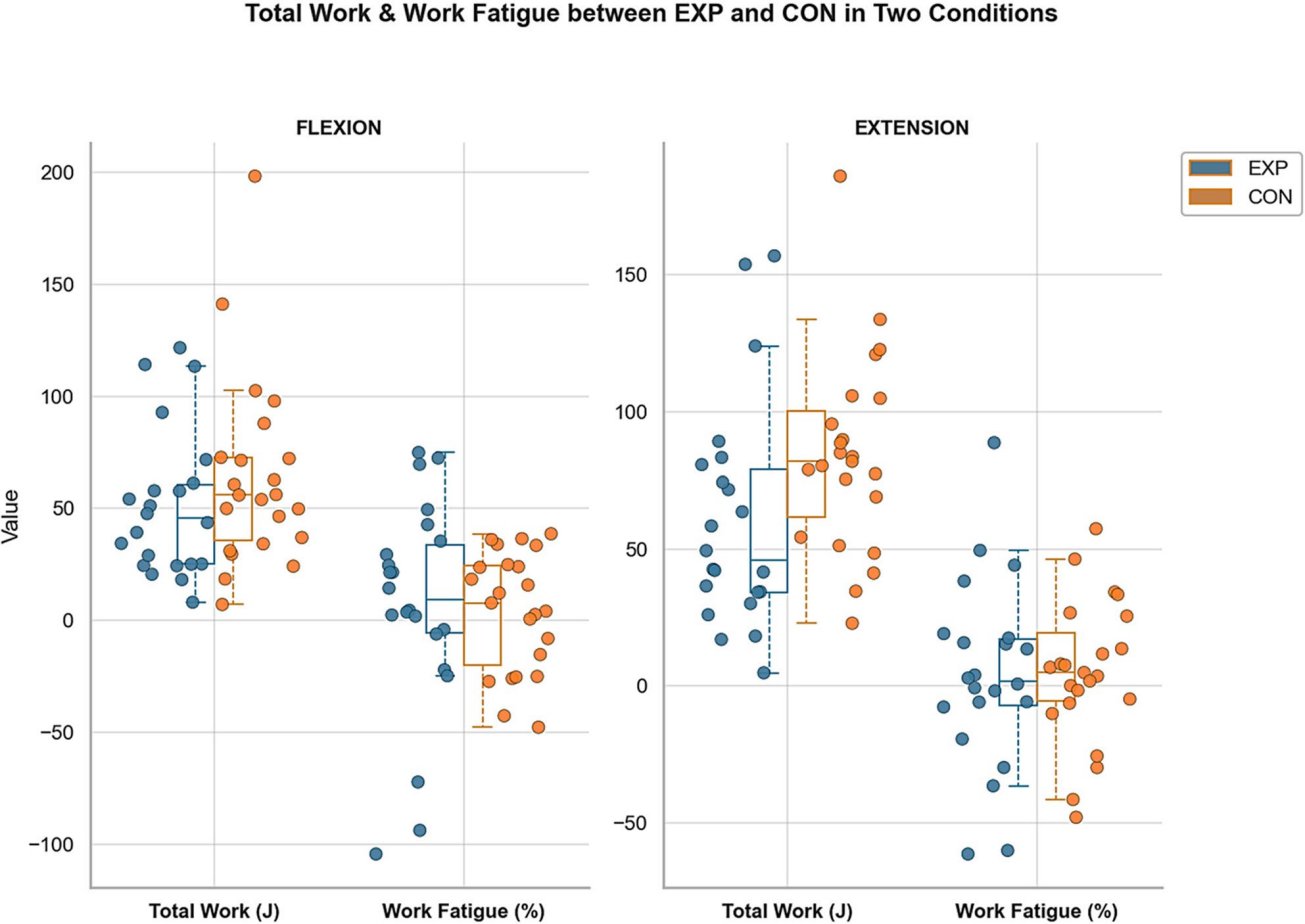
Total work and work fatigue output during wrist flexion and extension for both groups. Note: Flexion (FLEX), extension (EXT), students with chronic non-specific neck pain (EXP) and asymptomatic controls (CON)

Analysis of total work revealed no statistically significant differences between groups for either flexion or extension movements. In the extension condition, the asymptomatic control group generated 23.42 J more work than the experimental group, representing a borderline between-group difference (*p* = 0.050). Within-group comparisons indicated no significant difference between flexion and extension in either group. However, a trend toward greater work production during extension compared with flexion was observed in the asymptomatic control group (MD = 20.45 J, *p* = 0.084) (Fig. 6).

### Work fatigue

Work fatigue indices showed no significant differences between students with chronic neck pain and asymptomatic controls during either flexion or extension movements. This finding suggests that muscle endurance capacity remains preserved in individuals with chronic non-specific neck pain, despite the presence of ongoing symptoms (Fig. 6).

Separate two-way analyses of covariance (ANCOVA) were conducted for each dependent variable to examine the effects of group and condition (flexion vs. extension) on wrist muscle performance, with BMI included as a covariate to account for the between-group difference (*p* = 0.045). Using a Bonferroni-adjusted significance level (α = 0.0125), no significant group or group × condition interactions effects were observed for any dependent variable after controlling for BMI (all *p* > 0.0125).

### Correlation between pain intensity and muscle performance

Exploratory Pearson correlation analyses were conducted within the experimental group only (*n* = 44) to examine relationships between neck pain intensity and muscle performance measures. Pain intensity was measured using the Numeric Pain Rating Scale (NPRS, range 0–10), and correlations were calculated between NPRS scores and each of the four performance measures (peak torque, average power, total work, work fatigue) in both flexion and extension conditions, yielding a total of eight correlation tests. These analyses were exploratory and not adjusted for multiple comparisons. Correlation coefficients were interpreted according to conventional guidelines: |r| < 0.30 (weak), 0.30–0.50 (moderate), > 0.50 (strong).

A moderate positive correlation emerged between pain intensity and peak torque during wrist flexion (*r* = 0.47, *p* = 0.03), indicating that students and interns reporting higher neck pain intensity demonstrated greater flexor muscle strength. This relationship was weaker and non-significant during wrist extension (*r* = 0.34, *p* = 0.12).

Other muscle performance measures, including average power, total work and work fatigue, did not show significant correlations with pain intensity scores in either movement condition. These findings suggest that the relationship between neck pain and wrist muscle function may be specific to flexor muscle strength rather than representing a generalized effect on all aspects of muscle performance.

**Fig. 7:**
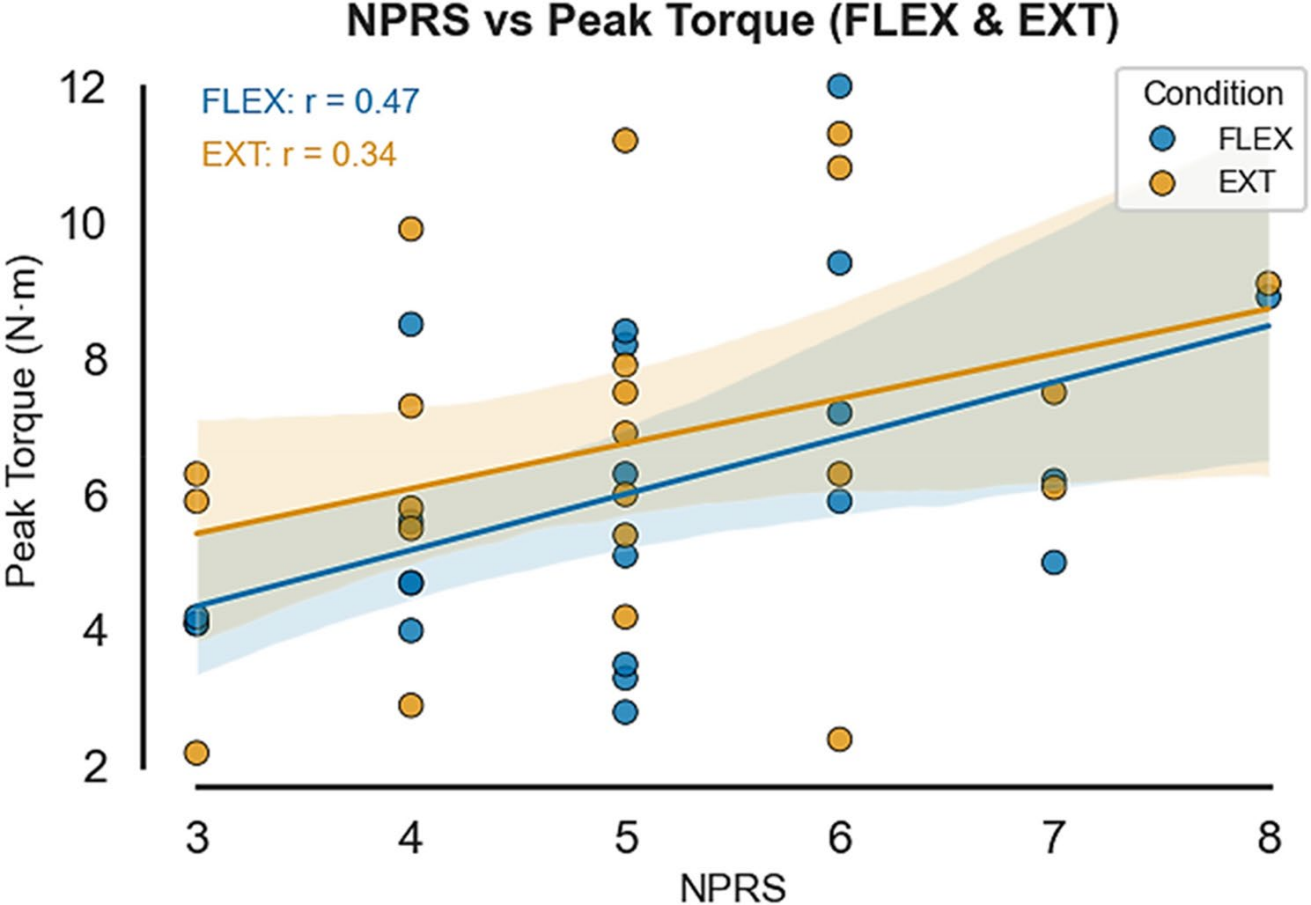
Exploratory correlation between neck pain intensity (NPRS, 0–10) and peak torque during wrist flexion/Extension in the CNSNP group (n = 44). A moderate positive correlation (r = 0.47, p = 0.03) indicates that students reporting higher neck pain intensity demonstrated greater flexor muscle strength. Note: Flexion (FLEX), extension (EXT)

The positive correlation between pain intensity and flexor strength may reflect compensatory neuromuscular adaptations or altered motor control patterns in response to chronic neck pain. This relationship warrants further investigation to understand its clinical implications and potential therapeutic targets (Fig. 7).

To ensure data integrity, especially given the high variance observed in endurance metrics, a rigorous data quality check was performed. All individual isokinetic torque curves were visually inspected post-testing. Trials were reviewed to exclude machine artifacts, baseline shifts, or invalid tests characterized by premature cessation of effort (e.g., a patient stopping completely before repetition 15). Extreme values were retained only if the continuous torque curve confirmed a valid, consistent effort by the participant.

## Discussion

The present findings indicate that wrist muscle performance during standardized concentric testing at 180°/s did not differ significantly between students with chronic non-specific neck pain (CNSNP) and asymptomatic controls. The multivariate analysis demonstrated no significant overall effect of group on the combined set of performance outcomes, suggesting that CNSNP was not associated with a generalized impairment in wrist isokinetic function.

In contrast, a significant multivariate main effect of movement condition was observed, indicating that wrist muscle performance differed between flexion and extension across participants, independent of group membership. This finding reflects a condition-related performance difference rather than a CNSNP-specific deficit. Interestingly, a task-specific adaptation linked to pain was introduced by the positive relationship between wrist-flexion peak torque and pain intensity; however, this association does not imply causation.

Contrary to earlier research that linked neck pain to upper-limb impairment, overall wrist performance in students and interns with CNSNP did not differ between groups [5, 10, 40]. This cohort has maintained general wrist power and endurance, according to standardized isokinetic testing; any deficiencies might be task specific.

Different results might occur at different velocities or during eccentric loading, where recruitment and fatigue behaviors differ, because performance was assessed using concentric contractions at 180°/s [41]. Pain-muscle relationships may be task-specific and velocity-dependent. Additionally, fifteen repetitions at this speed may not sufficiently challenge the neuromuscular system to detect subtle chronic-pain-related deficits in young, physically active physiotherapy students, and fatigue responses can vary across contraction types (dynamic vs. isometric sustained) [42].

The strict inclusion criteria regarding age (18–23 years) and BMI (19.5–29.5 kg/m²) were designed to minimize the influence of age-related neuromuscular decline. Consequently, the preserved motor function observed in this study may be attributed to the high physiological reserve of this young cohort, which could mask subtle impairments that might be more pronounced in older populations or those with metabolic comorbidities [43].

The cohort’s educational background ought to be considered. Participants may have been more knowledgeable about ergonomics, postural control, and self-management techniques because they were physiotherapy students and interns. This could enable compensatory movement techniques that lessen noticeable pain-related deficits during testing [44]. Limited exposure to sustained occupational loads, such as repetitive manual tasks or heavy lifting, may have reduced cumulative neuromuscular fatigue commonly observed in work-related musculoskeletal disorders. Although musculoskeletal complaints are prevalent among physiotherapy students and interns, those without substantial manual therapy or high-load practical exposure may retain better peripheral muscle function [45].

### Mechanical asymmetry between flexion and extension

In both groups, wrist extensor performance outperformed flexor performance, according to the statistically significant condition effect. Flexion peak torque (6.15 ± 2.72 N·m) was lower than extension peak torque (7.36 ± 2.56 N·m) (t (88) = 2.17, *p* = 0.033, d = 0.46). This pattern is consistent with established biomechanical expectations that, because of variations in muscle architecture (such as muscle mass, moment arm, and physiological cross-sectional area), wrist extensors frequently generate more torque than flexors [6, 33]. Additionally, altered co-activation strategies and greater reliance on superficial cervical musculature during limb tasks have been reported as effects of cervical dysfunction on upper-limb motor control [46].

In individuals with neck pain, the flexor-extensor mechanical imbalance may be assessed in the interpretation of functional findings. This represents greater force capacity in the extensors, potentially obscuring any pain-related reduction in output during standardized activities. However, the flexors were working closer to maximum and were more susceptible to even minor impairments with cervical pain or altered motor patterning. These findings are also consistent with reports that cervical pain-related neural adaptations can cause uneven effects on muscles within an upper-limb kinetic chain, resulting in changes specific to individual muscles and tasks rather than a generalized weakness [47].

### Positive correlation between pain intensity and flexor strength

A clinically meaningful and surprising relationship was detected between pain intensity (NPRS) and wrist-flexion peak torque (*r* = 0.47, *p* = 0.03). Rather than underperformance, this trend implies that individuals with CNSNP pain, having the greatest severity of pain, may have more flexor torque (and compensate at the muscle level).

This is consistent with research that links central sensitization and motor adaptation to chronic musculoskeletal pain, wherein persistent pain is accompanied by changes in motor control, such as greater synergist co-activation and protective muscle guarding [7, 48]. Increased flexor activation during wrist testing may therefore be a stabilizing, defensive approach in CNSNP, intended to increase proprioceptive input and joint stiffness, which may explain retained or even improved flexor torque despite persistent cervical discomfort. An explanation for these distal performance changes is that central sensitization may potentially alter cervical afferent transmission to the central nervous system, with subsequent consequences on peripheral sensorimotor function (including wrist proprioception) [49, 50].

On the other hand, this positive correlation found that a subset of students who experienced more pain and had a stronger neuromuscular drive or baseline fitness, enabling them to produce higher flexion peak torque despite their symptoms. The lack of significant correlations between pain intensity and the other isokinetic outcomes (total work, fatigue, and average power) provides proof that this effect is specific to flexion peak torque rather than reflecting a general improvement in wrist muscle function.

When evaluating the results of this study, it is important to consider several limitations. The larger age range should be included in future studies to better understand the effects of age-related changes on muscle function in CNSNP, since the limited age range (18–23 years) may not accurately reflect how aging affects muscle adaptation and performance. Selection students and interns may restrict generalizability, especially to occupational groups subjected to lengthy repeated work or greater physical burdens. To increase external validity, future research should include more varied samples from a variety of vocational backgrounds.

Unlike the general population, physiotherapy students and interns possess higher ergonomic awareness and health literacy due to their educational background. Additionally, their regular engagement in practical training implies higher physical activity levels. These factors may have served as protective mechanisms that attenuated pain-related impairments, meaning these findings cannot be extrapolated to sedentary office workers or older clinical populations.

Moreover, mild to moderate pain intensity on NPRS may mask effects found with severe levels of CNSNP. Moreover, cross-sectional design cannot determine causality between CNSNP and wrist performance changes.

Additionally, the study did not use electromyography (EMG) to measure muscle activation patterns, although an isokinetic dynamometer offers accurate, consistent measurements of muscle performance. Future research using EMG would enable a more thorough neuromuscular interpretation (e.g., altered recruitment, co-contraction, or compensatory activation of wrist and cervical muscles) and provided in the explanation of the mechanisms underlying motor behavior related to CNSNP.

In conclusion, the presence of CNSNP did not significantly impair overall wrist muscle performance during 180°/s concentric isokinetic testing in this sample of young adults. Rather than a global loss of force output, the observed increase in wrist flexor peak torque suggests a potential movement-specific adaptation in motor control.

#### Declaration of generative AI and AI-assisted technologies

During the preparation of this manuscript, the authors used generative artificial intelligence (Perplexity AI (version: December 2025)) tools to assist with language editing and improvement of clarity. The use of AI was limited to enhancing grammar, readability, and academic style. The AI tools were not used to generate scientific content, analyze data, interpret results, or draw conclusions. The authors carefully reviewed and edited all AI-assisted text and took full responsibility for the content of the manuscript.

## Data Availability

The datasets generated analyzed during the current study are available from the corresponding author on reasonable request.
